# The influence of six polymorphisms of uncoupling protein 3 (*UCP3*) gene and childhood obesity: a case–control study

**DOI:** 10.1186/s12887-023-03905-6

**Published:** 2023-02-21

**Authors:** Jakeline S. Fortes, Renata M. Pinto, Raissa F. de Souza, Fernanda R. Godoy, Raphael S. da Cruz, Daniela de M e Silva, Hugo P. Leite Filho, Aparecido D. da Cruz, Lysa B. Minasi

**Affiliations:** 1 Replicon Research Group, Genetics Graduate Program, School of Medical and Life Sciences, Pontifical Catholic University of Goiás, Rua 235, N. 40, Setor Leste Universitário, Goiânia, GO 74605-050 Brazil; 2 Genetics and Molecular Biology Graduate Program, Federal University of Goiás, Campus Samambaia, Goiânia, GO 74690-900 Brazil; 3 Pediatrics Department, Federal University of Goiás, Câmpus Colemar Natal E Silva (Câmpus I), Rua 235, Setor Leste Universitário, Goiânia, GO Brazil; 4 Physiotherapy Undergraduate Course, Centro Universitário de Goiânia – UNICEUG, Goiânia, GO Brazil; 5 Goiás State University, Ceres Campus, Ceres, GO Brazil; 6 Human Cytogenetics and Molecular Genetics Laboratory/CRER, State Health Secretary of Goiás, Goiânia, GO Brazil

**Keywords:** Insulin resistance, HDL, Z- BMI, Mitochondrial Uncoupling Protein

## Abstract

**Background:**

Obesity is defined as a multifactorial disease, marked by excessive accumulation of body fat, responsible for compromising the individual’s health over the years. The energy balance is essential for the proper functioning of the body, as the individual needs to earn and spend energy in a compensatory way. Mitochondrial Uncoupling Proteins (UCP) help in energy expenditure through heat release and genetic polymorphisms could be responsible for reducing energy consumption to release heat and consequently generate an excessive accumulation of fat in the body. Thus, this study aimed to investigate the potential association between six UCP3 polymorphisms, that have not yet been represented in ClinVar®, and pediatric obesity susceptibility.

**Methods:**

A case–control study was conducted with 225 children from Central Brazil. The groups were subdivided into obese (123) and eutrophic (102) individuals. The polymorphisms *rs*15763, *rs*1685354, *rs*1800849, *rs*11235972, *rs*647126, and *rs*3781907 were determined by real-time Polymerase Chain Reaction (qPCR).

**Results:**

Biochemical and anthropometric evaluation of obese group showed higher levels of triglycerides, insulin resistance, and LDL-C and low level of HDL-C. Insulin resistance, age, sex, HDL-C, fasting glucose, triglyceride levels, and parents’ BMI explained up to 50% of body mass deposition in the studied population. Additionally, obese mothers contribute 2 × more to the Z-BMI of their children than the fathers. The SNP rs647126 contributed to 20% to the risk of obesity in children and the SNP rs3781907 contribute to 10%. Mutant alleles of *UCP3* increase the risk for triglycerides, total cholesterol, and HDL-C levels. The polymorphism rs3781907 is the only one that could not be a biomarker for obesity as the risk allele seem to be protective gains the increase in Z-BMI in our pediatric population. Haplotype analysis demonstrated two SNP blocks (rs15763, rs647126, and rs1685534) and (rs11235972 and rs1800849) that showed linkage disequilibrium, with LOD 76.3% and D’ = 0.96 and LOD 57.4% and D’ = 0.97, respectively.

**Conclusions:**

The causality between *UCP3* polymorphism and obesity were not detected. On the other hand, the studied polymorphism contributes to Z-BMI, HOMA-IR, triglycerides, total cholesterol, and HDL-C levels. Haplotypes are concordant with the obese phenotype and contribute minimally to the risk of obesity.

## Background

Obesity is a global health issue, affecting people of different age groups, sexes, and economic status with two-thirds of the obese population living in developing countries [1, 2]. Increased adiposity is responsible for changes in body metabolism, fat mass distribution, and compromising several aspects of the individual health [3]. In addition, when obesity is a childhood outcome, it may be responsible for the development of life-threatening chronic diseases throughout their lives, which in turn contributes to increase public health expenditures adding a critical component to the financing of health systems [4].

Obesity is a complex multifactorial disease. Several factors, including lifestyle such as eating habits and social behaviors, metabolic individual heterogeneity, psychological disorders, neurological changes, and individual genetic makeup are involved in the origin and maintenance of this disease throughout life [5, 6]. It has been estimated that by the year 2025, approximately 2.3 billion adults worldwide will be overweight and that 1/3 of this number will be severely obese. In 2020, 39 million children under the age of 5 were overweight or obese [7]. Regarding pediatric obesity in Brazil, around 12.9% of children between 5 and 9 years old and 7% of the adolescents aged between 12 and 17 are already obese [8].

The fundamental cause underlying an obese phenotype is an imbalance between the amount of energy ingested compared to the amount of fat consumed by the body [5, 6, 9]. All consumed energy maintained in adipocytes is metabolized in the production of ATP or, in cases of excess, is alternatively eliminated by thermoregulation, producing heat. This burning through thermoregulation is only possible due to the presence of mitochondrial uncoupling proteins (UCP), formerly known as thermogenic. There are five isoforms of UCP in mitochondrial ridges, namely UCP1 to UCP5 [10].

The genes encoding the human UCP are studied in association with obesity, as several gene variants have been related to hypertension susceptibility, diabetes development, and cardiovascular diseases [11]. The *UCP3* gene, located at 11q13.4, has seven exons intervened by six intronic sequences. It is mainly expressed in both skeletal muscle and brown adipose tissue and the protein is related to cellular fatty acid metabolism. Upregulated expression of *UCP3* has already been related to physical activity, fasting, and high-fat diet. On the other hand, downregulation has been reported to improve fat oxidative capacity [12, 13]. When UCP3 activity is reduced, a decrease in energy expenditure is observed, while an increase in its expression is correlated with an increase in the metabolic rate, consequently, contributing to decrease BMI. The physiological function of UCP3 is yet unclear, nevertheless, it is found abundantly in brown adipose tissue (BAT) and may interplay with UCP1 in the regulation of BAT. Although several research groups have attempted to link UCP3 genomic variations with obesity, susceptibility in human and animal models remain conflicting and current trends show an increasing association of UCP3 with obesity and diabetes [14–16]

The gene variants most frequently used to investigate potential association with obesity susceptibility are Single Nucleotide Polymorphisms (SNP). To date, the risk alleles of about 750 loci of well recognized SNP with minimal allele frequencies as small as 1.6% have been contributing per-allele effects as low as 0.04 kg/m^2^ per allele [17]. Moreover, heritable factors appear to be responsible for up to 50% of the variation in body adiposity in non-syndromic polygenic obesity [6]. Different studies have been developed to observe the influence of genetic polymorphisms in the reduction of energy expenditure and, consequently, in the increase of fat deposition in tissues. Advancement on genome sequencing and genotyping has made it possible to investigate the genetic influence on the regulation of body fat. Thus, genomic variants have been associated with as high as 6% surplus on BMI when people carry the risk alleles [18]. Among *UCP3* polymorphisms, the frequently studied SNP are rs15763, rs1800849, rs647126, rs7930460, rs1685356, rs1685354, rs11235972, and rs378190719.

In the current study, a total of six *UCP3*’s SNP that have not yet been represented in ClinVar® – a public repository which aggregates information about genotype–phenotype correlation and its relationship to human health – were used to investigate the potential association between UCP3 genomic variation and pediatric obesity susceptibility. From the 6 SNP, 4 were categorized as mutations affecting gene transcription, namely rs1800849 (upstream variant), rs15763, rs647126, and rs1685354 (downstream variants) and 2 were in the class of intron variants, namely rs3781907 and rs11235972. The study group comprised of eutrophic and obese children from Central Brazil.

## Methods

### Study design and participants

The present observational case–control study included 225 children and adolescents, aged 5 to 19 years, of both sexes from Central Brazil. The participants were distributed as eutrophic or obese, corresponding to 102 and 123 people in each group, respectively. The study was performed following the ethical guidelines of the Declaration of Helsinki and was approved by the Ethics Committee on Human Research from the Pontifical Catholic University of Goiás under the registered number CAAE: 16303313.4.0000.0037. Written informed consent was voluntarily signed by parents/legal guardians of all participants.

Participants were recruited from the Endocrinology Service at the Children’s Hospital (Goiânia, Goiás, Brazil). Laboratory analyses were carried out at the genetic laboratory of Replicon Research Group/School of Medical and Life Sciences from the Pontifical Catholic University of Goiás and Mutagenesis Laboratory from Federal University of Goiás. Malnutrition, overweight, severe chronic diseases, genetic syndromes, and the use of medications that affected body weight and mass deposition were defined as exclusion criteria.

Anthropometric measurements including weight, height, and body mass index (BMI) of all participants and their biological parents were obtained by a pediatric endocrinologist with the aid of a fixed Tonelli E150-A stadiometer and a WELMY mechanical scale certified by the National Institute of Metrology, Quality and Technology (Inmetro). Inmetro is a governmental accreditation body responsible to maintaining the quality of instruments and pieces of equipment used in Brazil. The physician also performed a complete physical examination of the children and their biological parents. For all children, Z-scores of the BMI (Z-BMI) were calculated and interpreted according to the recommendation of the World Health Organization (WHO) [18]. Children were grouped according to the weight status as follows: obese Z-BMI ≥  + 2SD and eutrophic Z-BMI between < -2SD and + 1SD.

### Biochemical assays

Biochemical tests were performed in an accredited laboratory in Goiânia, Goiás, Brazil. Blood samples were drawn by peripheral venipuncture following a period of overnight fasting ranging from 8 to 12 h. The assays included fasting plasma glucose, lipid panel, and insulin levels. Total cholesterol (TC), triglycerides (TG), high-density lipoprotein cholesterol (HDL-C), low-density lipoprotein cholesterol (LDL-C), and glucose levels were run in an Abbott Architect c8000® Chemistry Analyzer. Insulin levels were determined by immunoassay using an automated Abbott Architect i2000®.

The reference values for fasting blood glucose are the same for adults and children. Thus, normoglycemic lays within the range of 3.3 and 5.6 mmol/L, pre-diabetics between 5.6 and 6.9 mmol/L and diabetics ≥ 7 mmol/L. Normal fasting insulin level is defined by the ranges 2.5 to 25 mIU/mL and < 15 mIU/mL for adults and children, respectively [19, 20]. To interpret the lipid panel, the recommendations of the Brazilian Society of Pediatrics [21] was used.

To assess insulin resistance in children, the HOMA-IR (*Homeostatic Model Assessment Insulin Resistance)* score was obtained using the equation: fasting serum insulin (μU/mL) x fasting plasma glucose (mmol/L)/22.5 [22]. The values obtained by calculating HOMA-IR, for the Brazilian child population, were interpreted through the parameters defined by [23].

### Genotyping *UCP3* polymorphisms

Genomic DNA was isolated from the peripheral blood of eutrophic and obese children using a commercial kit (AxyPrep™ BloodGenomic DNA Miniprep Kit, Axy353.gen Scientific, USA), following to the manufacture’s laboratory protocol. The concentration of genomic DNA was determined using a spectrophotometer (NanoVue Plus®, GE Healthcare, USA). Genotyping of the six SNP (*rs*15763, *rs*1685354, *rs*1800849, *rs*11235972, *rs*647126, and *rs*3781907) (Table 1) was carried out using qPCR (TaqMan® Real Time PCR in StepOnePlus™ thermocycler, Thermo Fisher Scientific, USA), following the manufacturer’s instructions.

**Table 1:** Probes sequences used for genotyping the *UCP3* gene polymorphisms in obese and eutrophic children from Central Brazil

SNP Id	Context Sequence (5′→3’)	VIC/FAM	Risk Allele^a^	Region
*rs*1800849	GGCTTGGCACTGGTCTTATACACAC**[A/G]**GGCTGACCTGAAACCTTATCCTAGA	A/G	A	5’ UTR
*rs*15763	TCCTTTGAGGTACTCATGATTGAGC**[A/G]**CGTGGTGGGGGGGGTGGGGAAGAGG	A/G	G	3’UTR
*rs*3781907	GAGCTCCACCTCTGGGGCAACCCCT**[G/A]**CCACATCCTGCCTGTGGTGTAGCTG	G/A	G	Intron
*rs*647126	CGCCAGCTCTGTGTTGCTGGGTGGC**[A/G]**TCCCTCCAGGCTCCGTGGCTGATCT	A/G	G	3’UTR
*rs*1685354	CCTTGTGGAGTTAGTCGTGCAGATT**[A/G]**GGTGAGTTAATGTTGGACAGCACTC	A/G	G	Intron
*rs*11235972	GGGCTGCCCCTGCAGCTTCCTTGAT**[A/G]**TCCACTCAGAGCCTCCTCATAAGCG	A/G	A	Intron

^a^The risk allele was assigned to the alternate allele provided in the dbSNP (https://www.ncbi.nlm.nih.gov/snp), which corresponded to the minor allele in the reference catalog

### Statistical analyses

Analyses were carried out in the SPSS® version 25.0 (IBM Corporation, USA) and Excel® version 16.62 (Microsoft Corporation, USA) utilities. In the current study, a significance level was set at 5% (*p* ≤ 0.05), considering confidence interval (IC) of 95%. Shapiro–Wilk’s and Levene’s tests were applied to check for normality and homogeneity of variance between groups before proceeding with additional analyses. Student’s t-test was used to compare the means of continuous variables between the studied groups. Pearson’s chi-square test (X^2^) was used to assess the deviations from Hardy‐Weinberg equilibrium and potential association between categorical variables, including the distribution of genotypes as a function of the risk allele between the obese and eutrophic groups. On the other hand, the standard McNemar test was used to determine if there were differences on the proportions of patients having wildtype alleles compared to the mutated genotype (risk alleles).

Multiple Linear Regression (MLR) was applied to observe whether the qualitative dependent variables could predict the outcomes of continues variables for the entire group. For the MLR the stepwise method was applied, and the equation was generated using beta standardized coefficients. Multicollinearity statistics was considered relevant to negatively affect the MLR model if tolerance was ≤ 20%. To test the assumptions’ compliances for MLR, the normality of the residues was verified according to the P-P plot graphs and the homoscedasticity by scatterplot. Furthermore, multinomial logistic regression (NR) was used to model the contribution of *UCP3* genotypes to the outcome variables. In SPSS, the dependent variable was ascribed to the aggregated genotypes containing the risk allele while the outcome variables (covariates) were Z-BMI, HOMA-IR, triglycerides, total cholesterol, and HDL-C. As the reference category, fasting glucose was used as their means were not statistically different between eutrophic and obese participants. Therefore, it would be possible to test the association for a dominant model, where the presence of a risk allele would contribute to modulate the outcome variables in the whole group of participants.

Haplotype block analysis was completed in Haploview® (Broad Institute) to test potential linkage disequilibrium (LD) for the studied SNP, using Gabriel’s confidence interval method [24]. The function multimarker haplotype tests block-specified, following the option to use logistic regression (GML), was performed for haplotype-based association analysis in Plink [25].

## Results

A total of 225 children and adolescents were enrolled in the study, including 123 (54.7%) and 102 (45.3%) participants in the obese and eutrophic groups, respectively. Regarding sex distribution, among the obese children, 60 (48.8%) were female and 63 (51.2%) were male, while in the eutrophic group, 52 (51%) were female and 50 (49%) males. Table 2 summarizes the demographic, anthropometric, and biochemical variables for both eutrophic and obese participants.

**Table 2:** Sociodemographic, anthropometric, and biochemical parameters for the group of obese and eutrophic children included in the study investigating the potential contribution of *UCP3* SNP variants on the susceptibility of pediatric obesity

Groups	Obese (*n* = 123)		Eutrophic (*n* = 102)		Student’s t test
Variables	**Means**	**SD**	**Means**	**SD**	***P***
Age (years)	9.7	2.5	10.1	2.6	0.192
Weight (kg)	55.6	20.0	30.4	10.0	< 0.001*
Height (cm)	142.6	13.9	135.2	15.6	< 0.001*
Z -BMI	3.1	1.0	-0.4	0.8	< 0.001*
Mother’s BMI (kg/m^2^)	29.6	6.3	24.3	3.3	< 0.001*
Father’s BMI (kg/m^2^)	32.5	6.8	26.9	4.6	< 0.001*
Glucose (mg/dL)	86.9	6.4	86.5	6.7	0.617
Insulin (µUl/mL)	12.9	7.1	6.3	2.8	< 0.001*
HOMA-IR	2.8	1.6	1.3	0.6	< 0.001*
TC (mg/dL)	169.6	29.7	162.3	26.3	0.052*
TG (mg/dL)	94.3	44.1	74.9	30.5	< 0.001*
HDL-C (mg/dL)	43.0	8.6	49.1	10.1	< 0.001*
LDL-C (mg/dL)	106.8	28.7	97.5	23.9	0.010*

^*^P ≤ 0.05 reveled statistical significance

It was observed that the mean values for weight, height, Z -BMI, mother’s and father’s, BMI, insulin levels, HOMA-IR, triglycerides, HDL-C, and LDL-C between obese and eutrophic groups were statistically different (p < 0.05). In the group of obese children, the mean value for HOMA-IR was 2.2 × higher than the mean of the eutrophic group, indicating insulin resistance among the obese children. Moreover, for the group of pediatric obesity, the mean of all variables revealed increased values but HDL-C, which showed an expected decrease. No statistical difference was observed for fasting glucose among the two groups. Table 2 summarizes those findings.

With respect to the normal distribution of data in the current study, few outliers were constant among all variables tested. However, Q-Q plots showed that skewness were observable only for the more extreme values related to outliers while most of the values for each variable remained adhered to the line of greatest fit as it would be expected if they truly came from a normal distribution. Consequently, because the data didn’t show heavy-tailed traits and because of the relatively large sample size of each group, non-normality presented no problems in hindering invalid further testing [26].

MLR was used to test if demographic, anthropometric, and biochemical independent variables could significantly predict the dependent Z-BMI for the whole pediatric population, consisting of obese and eutrophic participants. At first, it was identified and fixed multicollinearity among the predictors, which resulted in the removal of insulin (8,6%), height (13.4%), weight (14.7%), total cholesterol (15.6), and LDL-C (16.3%) from the model. Thus, the MLR results from the whole set of participants showed a statistically significant model (F = 347,4; *p* < 0.0001; *R*^2^ = 0.486), indicating that the predictors included in the model account for about 50% in the variability of Z-BMI in a pediatric population. The model only included in the equation variables that reached statistical significance:

$$Z-BMI=2.37+0.37HOMA-IR+0.3BMlm-0.25Age-0.18HDL-C+0.15Sex+0.13BMIf-0.12Glucose+0.04TG$$

The Pearson’s chi-square test showed the observed genotype distributions for the six SNP studied were not statistically significant different (p > 0.05) between obese and eutrophic groups. The chi-square test also showed the frequencies of the genotypes in the studied population complied with the Hardy–Weinberg equilibrium (p > 0.05). When evaluating the genotypic distribution as a function of the risk allele, no statistical differences were observed (Table 3). On the other hand, the McNemar test was applied to assess whether there was a difference between the proportions related to the presence of the risk allele by the obese phenotype. A statistically significant difference (p < 0.05) between the proportions of individuals who presented the risk allele and were eutrophic in relation to the obese individuals who presented wild-type alleles, with exception of the polymorphism rs647126 (Table 3).

**Table 3:** Allele and genotype distributions of six SNPs of *UCP3* of eutrophic and obese children from a pediatric population of Central Brazil

**SNP id**	**Group**	**Allele (Frequency)**		**McNemar**		**Genotype (Frequency)**			***Pearson’s Chi***	**Hardy–Weinberg Equilibrium**		
		**A (%)**	**G(%)**	**Chi**^**2**^	***P***	**AA (%)**	**AG (%)**	**GG (%)**	***P***	**Chi**^**2**^	***P***	**Comply**
***rs*1800849**	Eutrophic	36 (17.6)^a^	168 (82.3)	122.2	< 0.0001	2 (2.0)	32 (31.4)	68 (66.7)	0.265	0.642	0.725	Yes
	Obese	37 (15.1)^a^	209 (84.9)			3 (2.4)	31 (25.2)	89 (72.4)		0.023	0.988	Yes
***rs*15763**	Eutrophic	73 (35.8)	131 (64.2)^a^	10.8	0.001	11(10.8)	51 (50.0)	40 (39.2)	0.102	0.789	0.642	Yes
	Obese	83 (37.7)	163(66.3)^a^			14 (11.4)	55 (44.7)	54 (43.9)		0	1	Yes
***rs*3781907**	Eutrophic	145 (71.1)	59 (28.9)^a^	70.5	< 0.0001	50 (49.0)	45 (44.1)	7 (6.9)	0.504	0.544	0.762	Yes
	Obese	192 (78.0)	54 (22.0)^a^			76 (61.8)	40 (32.5)	7 (5.7)		0.319	0.852	Yes
***rs*647126**	Eutrophic	75 (36.8)	129(63.2)^a^	2.3	0.133	13 (12.7)	49 (48.0)	40 (39.3)	0.487	0.112	0.945	Yes
	Obese	106 (43.1)	140 (56.9)^a^			26 (21.1)	54 (43.9)	43 (35.0)		0.872	0.647	Yes
***rs*1685354**	Eutrophic	131 (64.2)	73 (35.8)^a^	33.7	< 0.0001	40 (39.2)	51(50.0)	11 (10.8)	0.106	0.789	0,674	Yes
	Obese	162 (65.9)	84 (34.1)^a^			53 (43.1)	56 (45.5)	14 (11.4)		0.019	0.991	Yes
***rs*11235972**	Eutrophic	36 (17.6)^a^	168 (82.4)	121.3	< 0.0001	2 (2.0)	32 (31.3)	68 (66.7)	0.716	0.642	0.725	Yes
	Obese	38 (15.4)^a^	208 (84.6)			3 (2.4)	32 (26.1)	88 (71.5)		0.002	0.999	Yes

^a^Risk allele

A nominal logistic regression (NLR) was performed to verify if the genotypes aggregated for the risk alleles in the *UCP3* gene were good predictors for the increase in the outcome variables. The multinomial logistic model was statistically significant (P ≤ 0,0001) for all six SNP included in the study. Table 4 includes all predictors that reached statistical significance in the model along with the betas and respective odds ratios. All SNP variants in *UCP3* were implicated in predicting the outcome variables in a pediatric population. Two SNP variants were able to predict positive changes in Z-BMI (rs647126 and rs3781907), two variants associated with positive changes to HOMA-IR (rs647126, rs15763) while, on the other hand, two had a negative impact on HOMA-IR (rs3781907 and rs1685354). Additionally, positive changes in total cholesterol could be predicted by two variant genotypes (rs1800849 and rs11235972) and one SNP associated with positive increase of HDL (rs3781907). Finally, two SNP variants associated with positive changes in triglycerides plasma concentrations.

**Table 4:** Logistic regression analysis when genotype was used as an independent variable and Z-BMI was taken as a dependent variable of participants of the study regarding the contribution of *UCP3* variants to obesity susceptibility in a pediatric cohort from Central Brazil

SNP ID	Covariates	Beta	OR	95% CI	*P*
**rs1800849**	Total Cholesterol	0.023	1.023	1,016—1.031	< 0.0001
***rs*647126**	Z-BMI	0.179	1.196	1.107—1.292	< 0.0001
	HOMA-IR	0.268	1.764	1.043—2.983	0.034
**rs15763**	HOMA-IR	1.409	4.092	1.842—9.089	0.001
	TG	0.007	1.007	1.003—1.010	< 0.0001
**rs3781907**	Z-BMI	0.099	1.104	1.053—1.157	< 0.0001
	HOMA-IR	-0.719	0.487	0.333—0.712	< 0.0001
	TG	0.007	1.007	1.004—1.009	< 0.0001
	HDL-C	0.030	1.031	1.021—1.040	< 0.0001
**rs1685354**	HOMA-IR	-1.314	0.269	0.121—0.596	0.001
**rs11235972**	CT	0.028	1.028	1.020—1.036	< 0.0001

Significance levels: *P* ≤ 0.05; *Beta* regression coefficient, *OD* Odds Ratio, *CI* Confidence interval

**Fig. 1:**
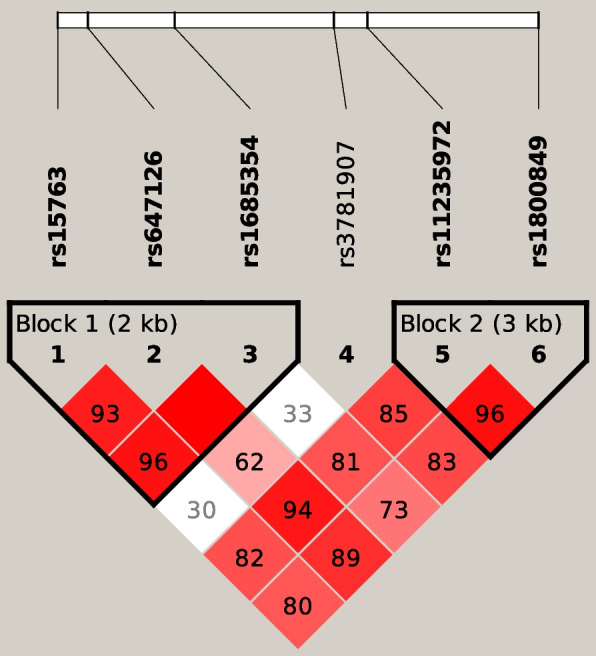
LD heatmap of region chr11: 74,000,400- 74,009,200, spanning over 8,800 bp, containing 6 SNPs in the *UCP3* gene. Figure Legend: The map depicts association signals achieved in a cohort of eutrophic and obese pediatric population from Central Brazil. Bright red signals depict pairwise linkage disequilibrium in the *UCP3* gene using six marker SNPs that associated with obesity in a pediatric cohort, indicating D’ ≥ 0.85 and LOD ≥ 3

With respect to haplotype frequencies, Fig. 1 showed the haplotypes on *UCP3* gene at 11q13.4 genotyped in association with pediatric obesity in a cohort of children and teens from Central Brazil. The first three SNPS (rs15763, rs647126, and rs1685354) were linked together in a separate haplotype block with a pairwise D’ value of 0.96, while a second block was inferred for SNP rs11235972 and rs1800849 with a pairwise D’ value of 0.97. Table 5 shows the results of plink/GML haplotype.

**Table 5:** Haplotype-based association analysis using Plink

NSNP	NHAP	CHR	BP1	BP2	SNP1	SNP2	Haplotype	F	OR	STAT	*P*
3	4	11	74000432	740025446	rs15763	rs1685354	AGG	0.338	1.09	0.185	0.667
3	4	11	74000432	740025446	rs15763	rs1685354	GGG	0.0112	0.8	0.0585	0.809
3	4	11	74000432	740025446	rs15763	rs1685354	GAA	0.395	0.758	2.1	0.148
3	4	11	74000432	740025446	rs15763	rs1685354	GGA	0.247	1.3	1.5	0.221
2	2	11	74006029	74009120	rs11235972	rs18000849	AA	0.158	1.13	0.226	0.634
2	2	11	74006029	74009120	rs11235972	rs18000849	GG	0.831	0.789	0.838	0.36

## Discussion

Understanding pediatric non-syndromic obesity requires the comprehension of the biological and environmental factors related to its development. Only after appreciating all the factors and covariables of such important heterogeneous, multifactorial, and complex disease that risk management could be planned to mitigate the health consequences for the susceptible population. For pediatric non-syndromic obese pollutions, clinical management has largely focused on metabolic concepts to cause or prevent severe outcomes. Scientific evidence has suggested that about 80% of obese children will become obese adults [27].

Genome-wide association studies (GWAS) with adult BMI and adiposity have associated many loci and thousands of variants with this phenotype. However, the contribution and role of those variants towards pathogenicity of obesity in adult and pediatric populations remains unanswered. Moreover, much less is known about the genetic variants associated with childhood obesity in the Brazilian population, well recognized for having extensive genetic admixture [28, 29]. The present study, although based on a discreet populational sample size, helps to contribute to the bulk of knowledge regarding the genetic influence of *UCP3* variants on the regulation of body fat deposition in an admixed pediatric population from Central Brazil.

In the current study, the sex of participants was not used as inclusion/exclusion criteria. Thus, random acceptance of both sexes led to the observation that pediatric obesity affected equally boy and girls. However, females seemed to be more susceptible to have larger Z-IMC than males as predicted by the Eq. 1.

Our findings showed a set of covariables that better explains the variability of the Z-BMI in the pediatric cohort. Insulin resistance, age, sex, HDL-C, fasting glucose, triglyceride levels, and parents’ BMI explained up to 50% of body mass deposition in the studied population. One relevant observation regarding the contribution of parent’s BMI to Children Z-IMC favors previous observations that the combination of genetic, environmental, and lifestyle factors, such as the obesogenic environment a child is exposed to, contributes to the obese phenotype and, consequently, undesired health outcomes [30, 31]. Additionally, obese mothers contribute 2 × more to the Z-BMI of the children than their fathers. With respect to risk mitigation, it’s important to clinicians and health support professionals to understand that complex traits are dependent on both genotypes and environment. Thus, children health outcomes are influenced by their parent’s habits during child growth and if a child shares genomic variants that even discretely increase their chances for obesity, wight managing must include a family plan. Being important to address and, whenever possible, modify parenting and lifestyles, habits, routine physical workouts, and modeling the home environment to reduce the risk of pediatric obesity [32–34]. It has been already postulated that when both parents are obese, the chance of a child developing obesity during childhood reaches 80%, however, when only one parent has the phenotype, the chance drops to 9% [35, 36].

The group of obese children showed higher levels of triglycerides, insulin resistance, and LDL-C and low level of HDL-C. Insulin resistance is related to the decrease of insulin capacity to uptake glucose [37] and it may contribute to becoming a risk group for the development of metabolic syndrome in children [38]. The accumulation of adipose tissue is responsible for triggering various physiological responses in the body, such as insulin resistance, increased cholesterol, and elevated blood pressure in those having the phenotype [39, 40]. Furthermore, adipose tissue is responsible for directly influencing the metabolism of glucose in the body [37–41] and is often related to Type 2 Diabetes Mellitus (T2DM) [42]. Therefore, special attention must be given to obese children due to the risk of developing non-communicable diseases leading to undesired and life-threatening comorbidities.

In general, genomic variants in the *UCP3* gene are responsible for interfering in energy homeostasis and related to cellular fatty acid metabolism. In the current study, the studied *UCP3* polymorphisms showed the variants were not causative of pediatric obesity. The genotypic distribution of all 6 SNPS agreed with the ALFA database for the Latin America available at dbSNP/NCBI. On the other hand, the proportions between the risk allele were statically different between eutrophic and obese participants, except for the SNP rs647126. Thus, although the risk alleles were not causative, they contributed to the phenotype. The SNP rs647126 and rs3781907 did contribute to the increase of Z-BMI in our pediatric populations. The first contributed to about 20% to the risk of obesity in children while the latter contribute around 10%.

More importantly, the association between *UCP3* variants and HOMA-IR was found for 4 *UCP3* SNP variants, namely rs647126, rs15763, rs3781907, and rs1685354, suggesting another important contribution to the *UCP3* in obesity susceptibly due to insulin resistance and, consequently, higher blood sugar levels, that, in turn, it is a well stablished risk for T2DM. The findings from our study leads to future experimental designs to further understand the role of *UCP3* in the development of T2DM. Moreover, increases risk for triglycerides, total cholesterol, and HDL-C levels were also associated with *UCP3* mutant alleles. Although the increments were discrete, future studies would be welcome to shed some light into the role of *UCP3* and cholesterol metabolism in humans (Table 4).

The association of these polymorphisms with overweight, obesity, and BMI has remained controversial. Different studies suggest the wild genotype of *rs*1800849 polymorphism is related to high levels of HDL-C, reduced chance to develop T2DM and decrease of BMI in adults [43–46]. A study showed no association between *rs*1800849 and BMI among Caucasian southern Italian children [10]. A study with Mexican adolescents did not show an association with BMI but showed a statistically significant difference with a lower waist circumference [47]. On the other hand, two other studies using Brazilian and French cohorts of adults demonstrated association of mutant alleles and T2DM [34, 48]. A Chinese study with obese adolescents did not find a significant association with rs1800849 gene polymorphisms and obesity [45].

Less is known about the rs11235972, rs647126, and rs15763 polymorphisms. A study by van Abeelen et al. reported an association between the mutant allele of the rs11235972 polymorphism and increased levels of CT and LDL-C [49]. The rs647126 polymorphism has been correlated with the increase of BMI in adult populations [49, 50]. The previous study did not observe a significant association between the rs15763 polymorphism and weight gain [51]. Additionally, there were reports of the relationship between mutant alleles of the rs1685354 polymorphism with the increase in BMI and consequently in the propensity of obesity development [49].

In the current study, rs3781907 polymorphism was the only one that could not be a biomarker for obesity as the risk allele seem to be protective against the increase in Z-BMI in a pediatric population (Table 4), despite the significant differences on the proportions of obese children having the risk alleles when compared to the wildtype genotype. Another study observed the subjects with the rs3781907-G allele had higher levels of CT and LDL-C and a higher risk of T2DM [51]. On the other hand, another study with a Chinese population did not associate this polymorphism with T2DM [50] nor with essential hypertension [52]. Further studies with obese children are still needed to elucidate the role of rs3781907 as a biomarker of obesity or related comorbidities in both pediatric and adult populations.

Finally, Fig. 1 depicts the association for all the genotyped *UCP3* SNP in association with pediatric obesity in a highly admixed Brazilian pediatric population. The X axis indicates the relative physical position of each SNP within the gene. The haplotype block map for the whole span of the *UCP3* gene, showing pairwise LD in D′, could be found in Table 6, which also included LOD and r^2^. Haplotypes and haplotype frequencies were shown in Table 7 for each defined haplotype block. The SNP blocks 1 (rs15763, rs647126, and rs1685534) and 2 (rs11235972 and rs1800849) showed linkage disequilibrium, indicating deviation of haplotype frequencies from expected values based on genotype frequencies. Thus, evidencing and observed degree of concordance of a *UCP3* variants with an obesity in a cohort of children from Central Brazil.

**Table 6:** Linkage disequilibrium scores to estimate the degree of concordance of *UCP3* SNP and pediatric obesity form a cohort of Central Brazil

SNP	rs647126	rs1685354	rs3781907	rs11235972	rs1800849	
rs15763	0.93	0.96	0.31	0.82	0.80	D’
	21.4	76.3	0.87	3.64	3.18	LOD
	0.31	0.91	0.02	0.07	0.07	r^2^
rs647126		1	0.62	0.95	0.89	D’
		27.8	5.1	9.3	7.90	LOD
		0.36	0.09	0.12	0.10	r^2^
rs1685354			0.33	0.82	0.74	D’
			0.99	3.48	2.89	LOD
			0.02	0.07	0.06	r^2^
rs3781907				0.86	0.84	D’
				24.7	22.7	LOD
				0.43	0.40	r^2^
rs11235972					0.97	D’
					57.4	LOD
					0.92	r^2^

*LD* Linkage disequilibrium, *D*’ disequilibrium coefficient

**Table 7:** Haplotype frequencies from 6 SNP distributed within the *UCP3* gene in a cohort of participants of a study on pediatric obesity from Central Brazil

Blocks	SNP id	Haplotype	Frequency (%)	Case ratios	Control ratios	Chi^2^	*P*
Block 1	rs15763 rs647126 rs1685354	GAA	39.5	73:131	105:141	2.220	0.1362
		AGG	33.8	71:133	81:165	0.176	0.6752
		GGA	24.7	56:148	55:191	1.557	0.2122
		GGG	1.1	2:202	3:243	0.057	0.8110
Block 2	rs11235972 rs1800849	GG	83.1	166:38	208:38	0.804	0.370
		AA	15.8	34:170	37:209	0.222	0.6377

The discrete population size may be considered a limitation for the current study, even though participants were recruited following precise selection criteria for both obese and eutrophic groups. At the same time, the lack of research to understand the biological role of *UCP3* genetic variants on cohorts of obese Brazilian children make the current study innovative in the field. Finally, another limitation of our study relates to the lack of assessment regarding the potential obesogenic environment shared by the participant children and their parents.

In conclusion, the present study enrolling a cohort of admixed Brazilian children could not detected causality between the six *UCP3* SNP and obesity. However, the studied polymorphisms contribute to Z-BMI, HOMA-IR, triglycerides, total cholesterol, and HDL-C levels. Here we reported the presence of the risk alleles increased the chances of children having obesity and related comorbidities. Due to controversial findings about the role of *UCP3* polymorphisms and obesity, further studies with a larger sample size and participants of distinct ethnic groups are required to confirm the potential of *UCP3* variants to obesity in humans.

We also highlighted the importance of management and follow-up of obese children to prevent metabolic disorders and comorbidity complications, such hypertension, T2DM, and cardiovascular diseases. The influence of the parents’ BMI on the Z-BMI of obese children reinforces the need for a careful look of this health problem under the assumption that childhood obesity is related to both genetic and environment factors. Thus, non-syndromic obesity should be managed and surveilled as a familial disease. The mitigation of deleterious health effects requires a holistic approach to guide the family to reduce their chance of undesired obesity outcomes. Unfortunately, however, for most non-syndromic obesity cases, a prognosis or a specific medical treatment/conduct protocols based on the contribution of multiple low risk alleles is not yet available, largely, due to the lack of knowledge regarding the multiple gene interactions and pleiotropic effect subjacent to complex traits, such as human obesity.

## Data Availability

The data used in this study are available from the corresponding author and could be requested by email. All SNP information are available at the links: https://www.ncbi.nlm.nih.gov/snp/rs1800849; https://www.ncbi.nlm.nih.gov/snp/rs15763; https://www.ncbi.nlm.nih.gov/snp/rs3781907; https://www.ncbi.nlm.nih.gov/snp/rs647126; https://www.ncbi.nlm.nih.gov/snp/rs1685354; https://www.ncbi.nlm.nih.gov/snp/rs11235972.

## Abbreviations

UCP3: Uncoupling Protein 3
DM2: Type 2 Diabetes Mellitus
SNP: Single Nucleotide Polymorphism
BMI: Body Mass Index
OR: Odds Ratio
HOMA-IR: Homeostatic Model Assessment Insulin Resistance
TC: Total cholesterol
TG: Triglycerides
HDL-C: High-density lipoprotein cholesterol
LDL-C: Low-density lipoprotein cholesterol
